# Assessment of the hypervascularized fraction of glioblastomas using a volume analysis of dynamic susceptibility contrast-enhanced MRI may help to identify pseudoprogression

**DOI:** 10.1371/journal.pone.0270216

**Published:** 2022-10-13

**Authors:** Margaux Roques, Isabelle Catalaa, Magali Raveneau, Justine Attal, Aurore Siegfried, Jean Darcourt, Christophe Cognard, Nicolas Menjot de Champfleur, Fabrice Bonneville

**Affiliations:** 1 Department of Neuroradiology, Toulouse Hospital, Toulouse, France; 2 Department of Radiotherapy, IUCT Toulouse (Toulouse University Cancer Institute), Toulouse, France; 3 Department of Pathology, IUCT Toulouse, Toulouse, France; 4 Department of Neuroradiology, Montpellier Hospital, Montpellier, France; Technische Universitat Munchen, GERMANY

## Abstract

**Purpose:**

Although perfusion magnetic resonance imaging (MRI) is widely used to identify pseudoprogression, this advanced technique lacks clinical reliability. Our aim was to develop a parameter assessing the hypervascularized fraction of glioblastomas based on volume analysis of dynamic susceptibility contrast-enhanced MRI and evaluate its performance in the diagnosis of pseudoprogression.

**Methods:**

Patients with primary glioblastoma showing lesion progression on the first follow-up MRI after chemoradiotherapy were enrolled retrospectively. On both initial and first follow-up MRIs, the leakage-corrected cerebral blood volume (CBV) maps were post-processed using the conventional hot-spot method and a volume method, after manual segmentation of the contrast-enhanced delineated lesion. The maximum CBV (rCBVmax) was calculated with both methods. Secondly, the threshold of 2 was applied to the CBV values contained in the entire segmented volume, defining our new parameter: %rCBV>2. The probability of pseudoprogression based on rCBVmax and %rCBV>2 was calculated in logistic regression models and diagnostic performance assessed by receiving operator characteristic curves.

**Results:**

Out of 25 patients, 11 (44%) were classified with pseudoprogression and 14 (56%) with true progression based on the Response Assessement in Neuro-Oncology criteria. rCBVmax was lower for pseudoprogression (3.4 vs. 7.6; p = 0.033) on early follow-up MRI. %rCBV>2, was lower for pseudoprogression on both initial (57.5% vs. 71.3%; p = 0.033) and early follow-up MRIs (22.1% vs. 51.8%; p = 0.0006). On early follow-up MRI, %rCBV>2 had the largest area under the curve for the diagnosis of pseudoprogression: 0.909 [0.725–0.986].

**Conclusion:**

The fraction of hypervascularization of glioblastomas as assessed by %rCBV>2 was lower in tumours that subsequently developed pseudoprogression both on the initial and early follow-up MRIs. This fractional parameter may help identify pseudoprogression with greater accuracy than rCBVmax.

## Introduction

Pseudoprogression diagnosis during follow-up of treated glioblastomas remains an important issue in neuro-oncology and, in many cases, a diagnostic challenge. Its clinical definition often varies between studies but is classically described as an increasing contrast-enhanced lesion on the 1^st^ follow-up magnetic resonance imaging (MRI), mimicking tumour progression, that subsequently stabilises or decreases in size without additional treatment [1, 2]. There is no consensus on morphological features or quantitative values of any advanced MRI technique to establish diagnosis of pseudoprogression [3–5]. On the other hand, the histopathological diagnosis, invasive by nature, can suffer from sampling bias and complex interpretation [6]. Hence, pseudoprogression diagnosis is usually possible retrospectively on the subsequent MRI, performed 1 to 2 months later, if it demonstrates stabilisation or regression of the enhancing lesion. This leads to a gap before adequate treatment is started, which can be damageable for patients with a grim prognosis [7].

Dynamic susceptibility contrast-enhanced MRI (DSC-MRI) is considered the method of choice to distinguish radiation effect from tumour progression by the majority of European institutions [8]. Neoplasm induces the formation of pathological new vessels, indirectly assessed by an increase in the relative cerebral blood volume (rCBV), whereas theoretically radiation-induced lesions do not. However, there is considerable variability in published rCBV thresholds for tumour recurrence, ranging from 1.5 to 3 [9]. These discrepancies may result from diverse MRI-perfusion techniques such as the use of a pre-load of gadolinium or different post-processing methods [10]. Additionally, the significant heterogeneity that characterises glioblastomas and pseudoprogression can be a significant drawback [11, 12]. The conventional and widely used post-processing method consists in manually drawing regions of interest (ROI) in selected enhancing and hypervascularized “hot-spot” of the tumour to assess the maximal rCBV of the tumour (rCBVmax). This technique inevitably leads to sampling bias and substantial inter- and intra-observer variability [13, 14]. However, to date, no alternative has been proposed in clinical practice and these limits are still accepted by the medical community.

For better consideration of the heterogeneity of glioblastomas, some authors have suggested analysing MRI-perfusion over the entire volume of the enhancing portion. CBV value histogram analysis has already shown greater diagnostic accuracy and inter-observer agreement in glioma grading [15, 16]. Applied to treated glioblastomas, changes in the histographical pattern of CBV values during follow-up was an excellent independent predictor of early tumour progression [17]. However, the study could not identify a predictive factor of pseudoprogression on the early follow-up MRI, when an increase in the gadolinium-enhanced lesion is observed and pseudoprogression is suspected. In addition, these techniques can appear tedious and parameters such as kurtosis or skewness seem abstract or non-intuitive to radiologists, thus remaining limited to a research setting.

The aim of our study was to develop a volume analysis of DSC-MRI, more exhaustive than the hot-spot method, without visual a priori and accessible in routine clinical practice, to assess the hypervascularized fraction of cerebral tumour and evaluate its performance in the diagnosis of pseudoprogression of treated glioblastomas.

## Materials and methods

### Study population

**Fig 1:**
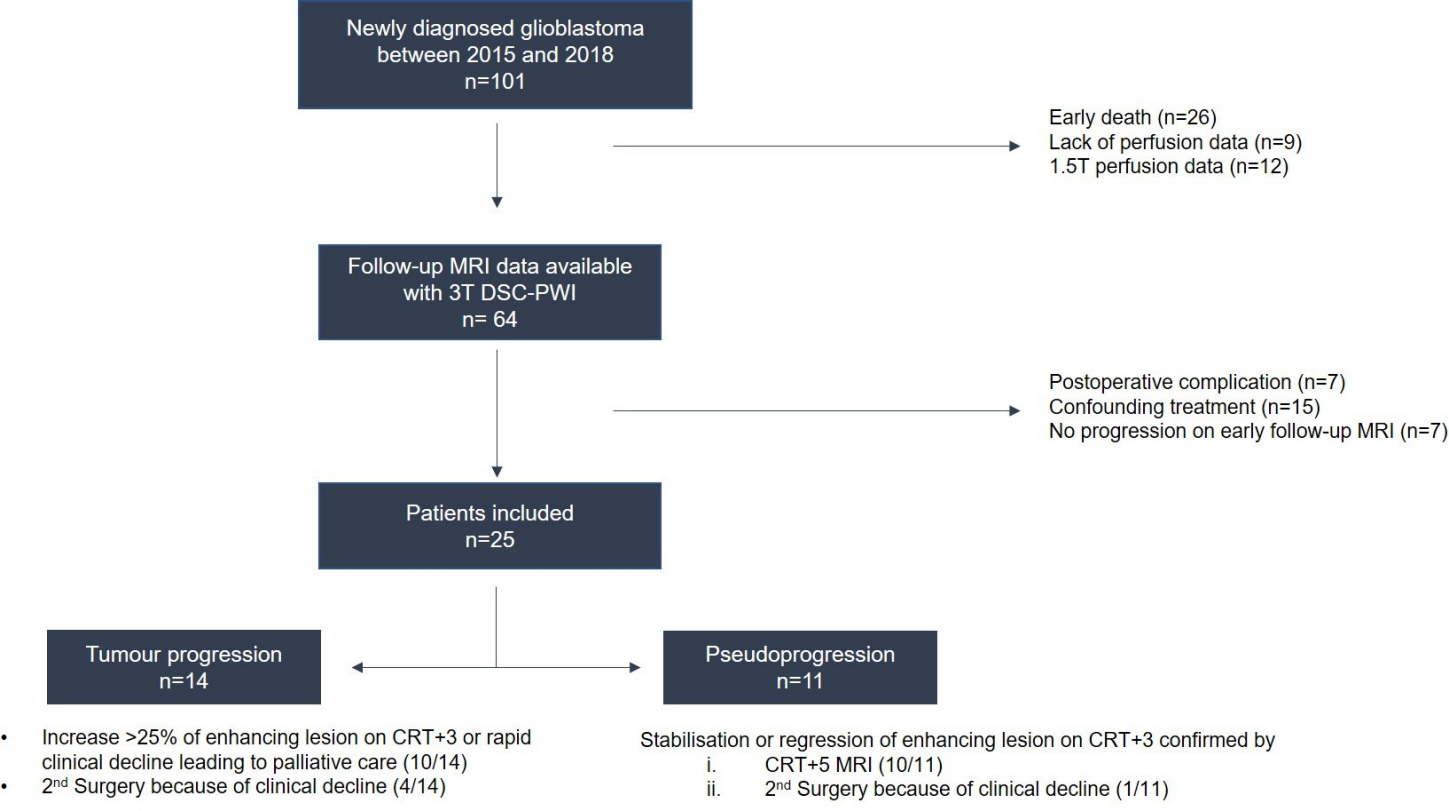
Flow-chart. Twenty-five patients with pathologically proven primary glioblastoma, with at least 4 MRIs and usable perfusion data acquired on 3T systems, treated by chemoradiotherapy showing progressive lesion enhancement on early follow-up MRI (CRT+1) were included.

This retrospective study was approved by our institutional review board (CRM-1906-012). One hundred and one consecutive patients with primary glioblastoma were identified from our database over a 4-year period. They were included if they had undergone: (a) chemoradiotherapy (CRT) after surgical resection or biopsy, (b) 4 MRIs: an initial MRI at the time of the diagnosis, an immediate postoperative MRI 24-72h after surgery, an early follow-up MRI, performed in our centre 1 month after CRT completion (CRT+1) and a subsequent follow-up MRI, 3 months after CRT completion (CRT+3), (c) DSC-MRI on a 3T system, (d) progressive contrast-enhanced lesions on the CRT+1. Among these patients, 76 were excluded and the remaining 25 constituted our cohort (Fig 1). For each case, patient characteristics included age, gender, extent of resection, initial WHO performance status, initial tumour volume, irradiation dose, molecular markers (IDH, MGMT) and overall survival (referred as the time between the date of initial MRI and the date of death or date last known alive).

### Identification of pseudoprogression versus tumour progression

Two senior neuroradiologists (M.R and I.C) assessed MRI images according to the Response Assessement in Neuro-Oncology criteria (RANO) [18]. Among the 25 patients who demonstrated radiological progression between postoperative MRI and CRT+1, those with stabilisation or regression (≥ 50%) of their enhancing lesion on the following CRT+3 MRI were considered to have pseudoprogression, confirmed by either a 2^nd^ subsequent MRI or histopathologic assessment (n = 4). Patients with any new enhancing lesion outside of the radiation field, an increase ≥ 25% in enhancing lesions or rapid clinical decline leading to palliative care, or secondary surgical resection with histopathologic confirmation (n = 1), were considered to have tumour progression.

### MRI parameters

Images were acquired using two 3T systems *(Magnetom Skyra*, *Siemens Healthcare and Achieva*, *Philips Medical System*). The imaging protocol included at least axial spin-echo T1-weighted imaging, axial fluid-attenuated inversion recovery imaging (FLAIR), followed by DSC-MRI data and contrast-enhanced gradient-echo 3D T1-weighted imaging (CE-T1WI).

DSC-MRI was acquired with a gradient-echo echoplanar imaging technique during the first pass of a standard bolus of gadolinium contrast without pre-load bolus. The imaging parameters were as follows: *Magnetom*: TR 1710 ms, TE 20 ms, slice 4 mm, flip angle 90°; *Achieva*: TR 1657 ms, TE 40 ms, slice thickness 4 mm, flip angle 75°. During 45 consecutive echoplanar imaging scans lasting 1 minute 30 seconds, an intravenous bolus injection of 0.2 ml/kg gadolinium chelate (Gadobenate dimeglumine, *Multihance®*, *Bracco*, *Italy* or Gadoteric acid, *Dotarem®*, *Guerbet*, *France*) was administered at a flow rate of 5 ml/s followed by a 20 ml saline flush.

3D CE-T1WI data were acquired with the following parameters for *Magnetom*: TR 1670 ms, TE 2.30 ms, slice thickness 1 mm, flip angle 8° and *Achieva*: TR 9.89 ms, TE 4.60 ms, slice 1 mm, flip angle 8°. FLAIR data were acquired with the following parameters for both machines: TR 8000 ms, TE 100 ms, slice thickness 3 mm.

### DSC-MRI data post-processing

**Fig 2:**
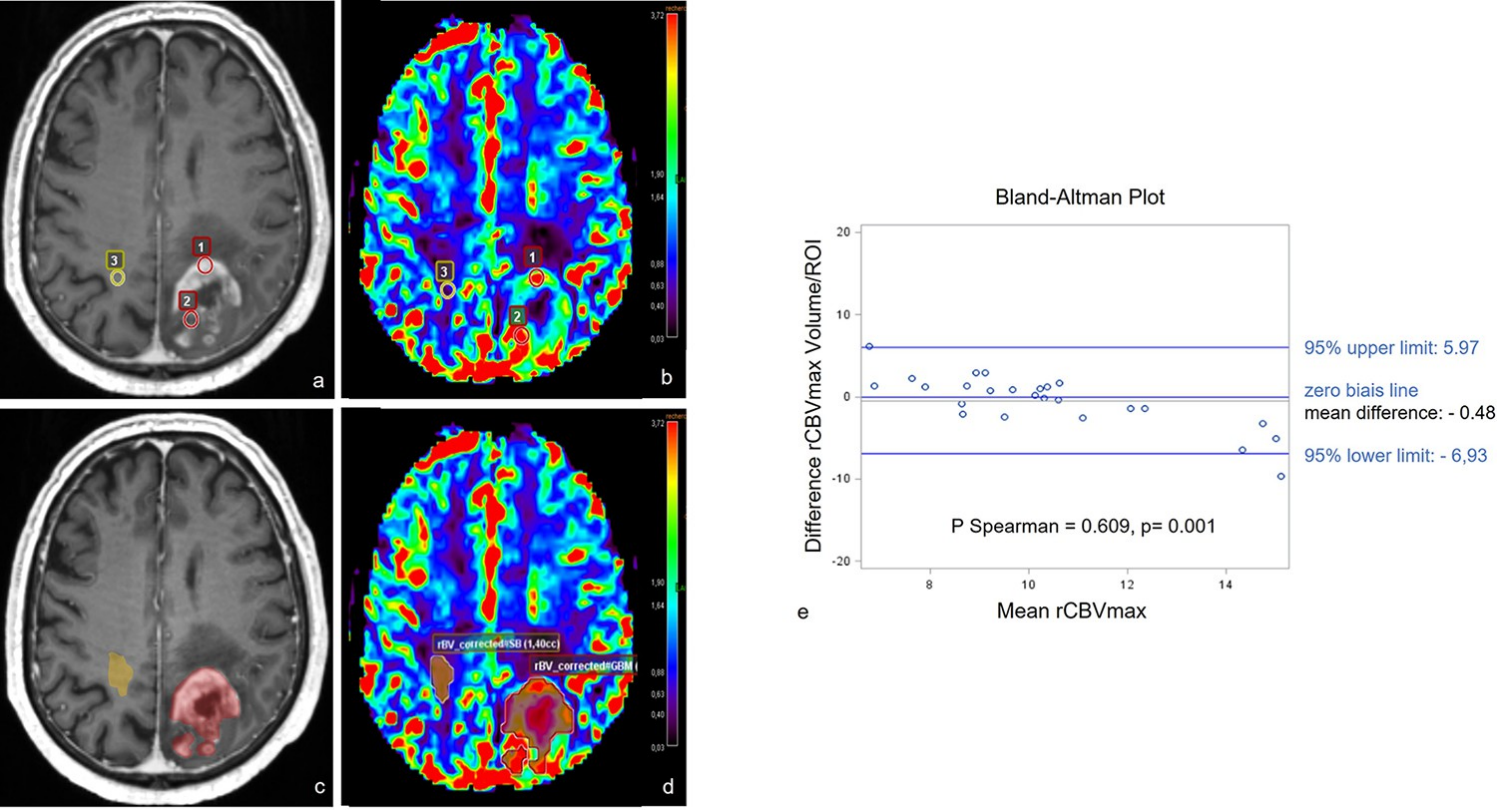
Illustration of the two different methods. Two methods were used to calculate the maximum relative cerebral blood volume (rCBVmax) and %rCBV>2, in a 60-year-old patient with glioblastoma. a) and c) axial contrast-enhanced T1-weighted images; b) and d) corrected rCBV maps. Top line, the hot-spot method: Two ROIs (red) are placed in hypervascularized areas of the enhancing tumour and one (yellow) is placed in the contralateral white matter for normalization. Bottom line, the volume method: Segmentation of the enhancing tumour (red) slice by slice including areas of high and low neoangiogenesis and segmentation of a volume of interest in the contralateral white matter (yellow) for normalization. e) The Bland-Altmann plot illustrates the concordance between the two methods used to analyse rCBVmax.

DSC-MRI data acquired on both initial and CRT+1 MRI were post-processed by a neuroradiologist expert in oncology, as illustrated in Fig 2, with a constructor-independent commercial software, using a unidirectional leakage correction algorithm for calculation of corrected CBV maps (*Olea Sphere 3*.*0 SP-6*, *Olea medical*, *La Ciotat*, *France)*.

First, using the hot-spot method of Wetzel et al which showed the best inter/intra-observer reproducibility [19]. A single observer manually delineated 3 to 4 pre-shaped circular ROIs, ranging from 40 to 60mm^2^, in the enhancing lesion after visual assessment of CBV maps and normalised it with normal contralateral white matter to define rCBV. The highest rCBV value obtained among these ROIs was considered as rCBVmax.

Secondly, a volume analysis was performed as follows: 1) manual segmentation, of the contrast-enhanced delineated lesion on the 3D CE-T1WI images, slice by slice, including central necrosis; 2) volume masks were transferred to the CBV maps using a rigid 3D co-registration algorithm; 3) the maximal CBV value of the segmented volume was automatically generated by the software; 4) normalisation was performed by delineation and calculation of the mean CBV of 3 freehand ROIs placed in the contralateral white matter at the superior, middle and inferior levels of the tumour and 5) CBV values of each pixel contained in the volume were extracted for analysis. The new parameter %rCBV>x was defined by the fraction of pixels of the segmented volume containing values above various thresholds (x) ranging from 1.5 to 3.

To assess the reproducibility of this original method, a second neuroradiologist (M.Ra.) independently calculated this new parameter on early follow-up MRI data, acquired 1 month after CRT.

Finally, on CRT+1 MRI, the volume analysis was performed a second time, manually excluding central necrosis from the segmented volume, slice by slice.

### Statistical analysis

Patient characteristics were compared between the groups with Fisher’s exact tests for categorical data and Mann-Whitney-Wilcoxon tests for continuous variables.

The concordance between rCBV evaluation methods was assessed on the initial MRI, before any treatment was initiated, with Spearman’s correlation coefficient and Bland-Altman.

To determine the threshold (x) for which the %rCBV>x values are best to differentiate pseudoprogression from tumour progression, receiver operating characteristic (ROC) analysis with calculation of area under the curve (AUC) were carried out on the perfusion data obtained on the early follow-up MRIs (CRT+1). Seven different thresholds (1.5;1.75; 2; 2.25; 2.5; 2.75 and 3) were tested. The threshold value was considered optimal when the Youden index (Sensitivity + Specificity—1) reached a maximum. We hypothesized that %rCBV>2 could differentiate between pseudoprogression and tumour progression. rCBVmax and %rCBV>2 of both groups were compared using Mann-Whitney-Wilcoxon tests.

The probability of pseudoprogression based on rCBVmax measured by a) the hot-spot method, b) the volume method and %rCBV>2, on both initial MRI and CRT+1, were calculated in logistic regression models. The goodness of fit of the models was assessed by Hosmer-Lemeshow tests. Predictive performance was estimated using ROC AUCs and presented with their 95% confidence intervals [95% CI]. Sensitivity and specificity were calculated for cut-off values determined with the Youden index.

For inter-observer reproducibility, the intraclass correlation coefficient (ICC) of quantitative measurements of %rCBV>2 and a kappa score for pseudoprogression diagnosis were calculated. The ICC was calculated with a two-way random model, with single measure consistency and reported with their 95% confidence interval. Agreement was considered using standard guidelines [20].

All tests were bilateral and a p-value of 0.05 indicated statistical significance. Statistical analysis was performed with a biostatistician (A.G.) using commercially available software SAS Version 9.4 (SAS Institute Inc., Cary, NC, USA) and STATA version 14.2 (StataCorp LP, College Station, TX, USA).

## Results

### Patients

Among the 25 patients included, 11 were classified as having pseudoprogression (44%) and 14 as having tumour progression (56%). The entire cohort had a median age of 62 years at the time of diagnosis [range 42–82]. There were 11 women (44%) and 14 men (56%) and median survival was 17.1 months [5.4–47.5]. The patients in the pseudoprogression group were significantly younger (57 vs. 68.5 years; p = 0.01) and had a longer survival (26 vs. 14.5 months; p = 0.019) than patients in the true progression group. The MGMT promoter methylation status was positive in 55% of patients with pseudoprogression and in 36% of patients with tumour progression (p = 0.435). The basic patient characteristics are summarised in Table 1.

**Table 1:** Patient characteristics.

Characteristics	Cohort (n = 25)	Pseudoprogression (n = 11)	Tumor progression (n = 14)	p-value
Age [years]	62 [42–82]	57 [42–67]	68.5 [47–82]	*.01 ꝉ
Gender				1 ǂ
• Female	11 (44)	5 (46)	6 (43)	
• Male	14 (56)	6 (54)	8 (57)	
Surgery				.35 ǂ
• Subtotal resection	19 (76)	7 (64)	12 (86)	
• Gross Total resection	6 (24)	4 (36)	2 (14)	
WHO performance score				1 ǂ
• 0–1	20 (80)	9 (82)	11 (79)	
• >1	5 (20)	2 (18)	3 (21)	
Initial tumor volume (cc) *	43.9 ± 30.7	53.9 ± 31.9	36.1 ± 28.4	.171 ꝉ
Irradiation dose (Gy) *	59.7 ± 1.2	59.9 ± 0.2	59.4 ± 1.7	.258 ꝉ
IDH mutation status	1 (4)	1 (7.7)	0 (0)	.999 ǂ
MGMT methylation status (+:-:NA)	7:7:11	2:1:8	5:6:3	NA
Survival (months)°	17.1 [5.4–47.5]	26 [10.3–47.5]	14.5 [5.4–44.4]	*.019 ꝉ

Except where indicated, data are number of patients and numbers in parentheses are percentage

* Data are mean ± standart deviation and° Data are median and [range]

ꝉ Calculated with the non parametric Mann-Whitney-Wilcoxon test

ǂ Calculated with Fisher’s exact test

NA Non available data

### Concordance between the hot-spot and volume methods of assessing rCBVmax on the initial MRI

The median rCBVmax values of untreated glioblastomas calculated with the two different methods were comparable: 9.8 [8.9–10.8] with the hot-spot and 10.2 [9.6–10.7] with the volume method. The two post-processing methods had a good level of concordance: the mean difference between the calculated values was -0.48, with the highest variations for extreme values and the Spearman correlation coefficient differed significantly from 0 (ρ = 0.609; p = 0.001) (Fig 2).

### Differentiating between subsequent pseudoprogression and tumour progression based on parameters measured on the initial MRI

**Fig 3:**
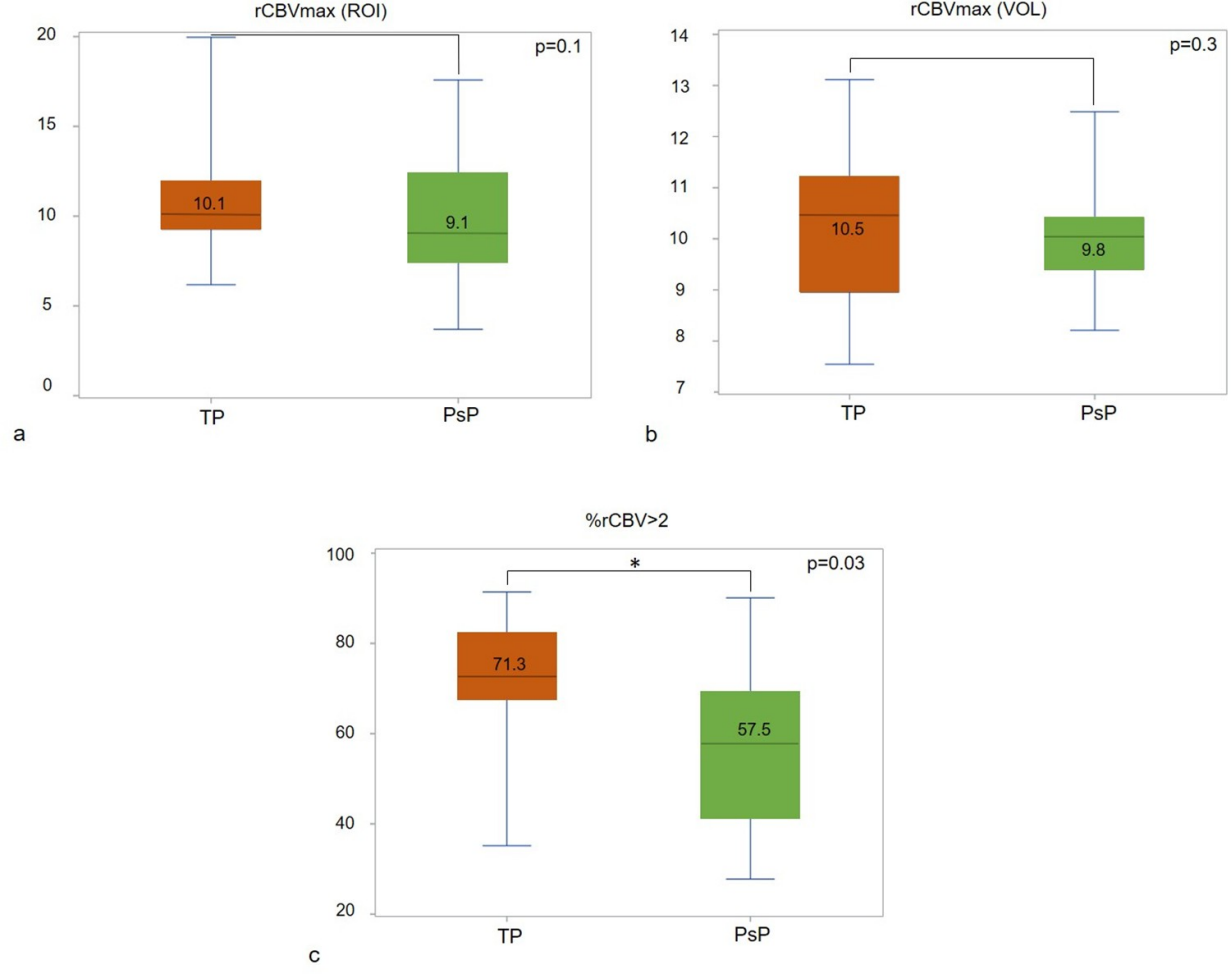
%rCBV>2 is the only parameter that distinguish tumour progression from pseudoprogression on the initial MRI. Box-and-whiskers graphs of the maximum relative cerebral blood volume (rCBVmax) on the initial MRI in the tumour progression (TP) or pseudoprogression (PsP) groups obtained with a) the hot-spot method (ROI), b) the volume method (VOL), and c) %rCBV>2. %rCBV>2 was the only significantly lower parameter in the PsP group.

On the initial MRI, with the hot-spot method, the median rCBVmax was not different between patients with subsequent pseudoprogression and tumour progression, 9.1 [7.1–12.4] vs. 10.1 [9.6–13.4] (p = 0.14), respectively. A similar finding was obtained with the volume method, namely 9.8 [8.7–10.6] vs. 10.5 [9.5–11.1] (p = 0.27), respectively (Fig 3A and 3B). The fractional perfusion parameter, %rCBV>2, was 63.9% ± 18.7 for the entire cohort. It was significantly lower for patients who developed pseudoprogression than for those with subsequent tumour progression, 57.5% [32.7–70.6] vs. 71.3% [66.8–80.3] respectively, (p = 0.03) (Fig 3C).

### Differentiating between pseudoprogression and tumour progression based on parameters measured on the early follow-up MRI

**Fig 4:**
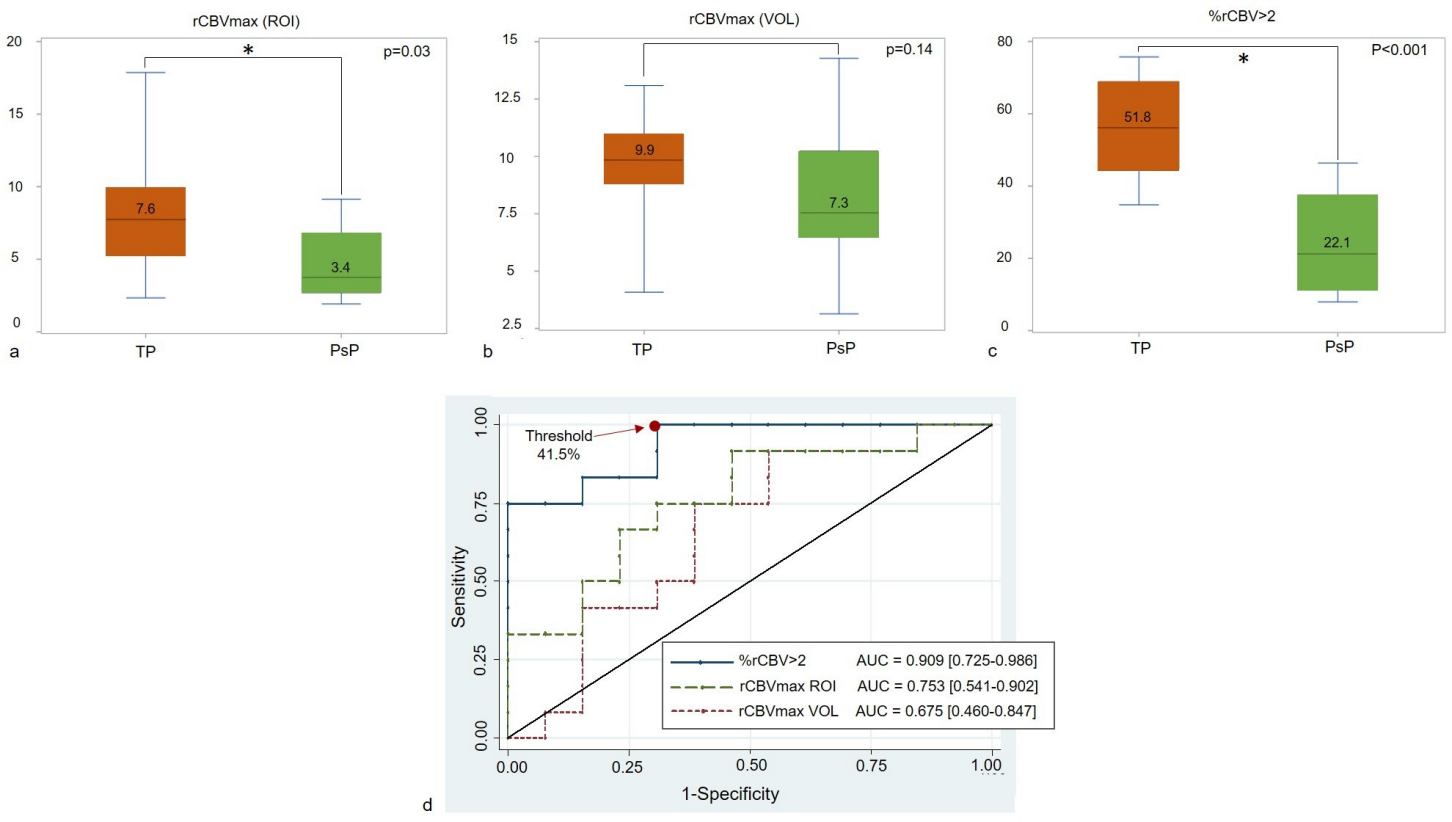
%rCBV>2 differentiate tumour progression from pseudoprogression on the early follow-up MRI with a higher confidence level than rCBVmax. Box-and-whiskers graphs of the maximum relative cerebral blood volume (rCBVmax) on early follow-up MRI (CRT+1) in the tumour progression (TP) or pseudoprogression (PsP) groups obtained with a) the hot-spot method (ROI), b) the volume method (VOL) and c) %rCBV>2. rCBVmax and %rCBV>2 were significantly lower in the PsP group. d) ROC curves for rCBVmax (ROI), rCBVmax (VOL) and %rCBV>2. Best Youden index for pseudoprogression diagnosis was obtained when %rCBV>2 was less than 41.5%.

On CRT+1 MRI, with the hot-spot method, rCBVmax was lower for pseudoprogression than for tumour progression, 3.4 [2.4–7] vs. 7.6 [4.9–9.6], respectively (p = 0.03) but with substantially overlapping values (Fig 4A). Calculated with the volume method, the rCBVmax value did not differ statistically between the two groups: 7.3 [5.12–10.6] vs. 9.9 [8.9–10.9] (p = 0.14) (Fig 4B). %rCBV>2 was 39.7% ± 21.5 for the entire cohort after CRT and was lower in the pseudoprogression group than in the tumour progression group: 22.1% [9.9–37.4] vs. 51.8% [39.6–64.6] (p <0.001) (Fig 4C).

**Fig 5:**
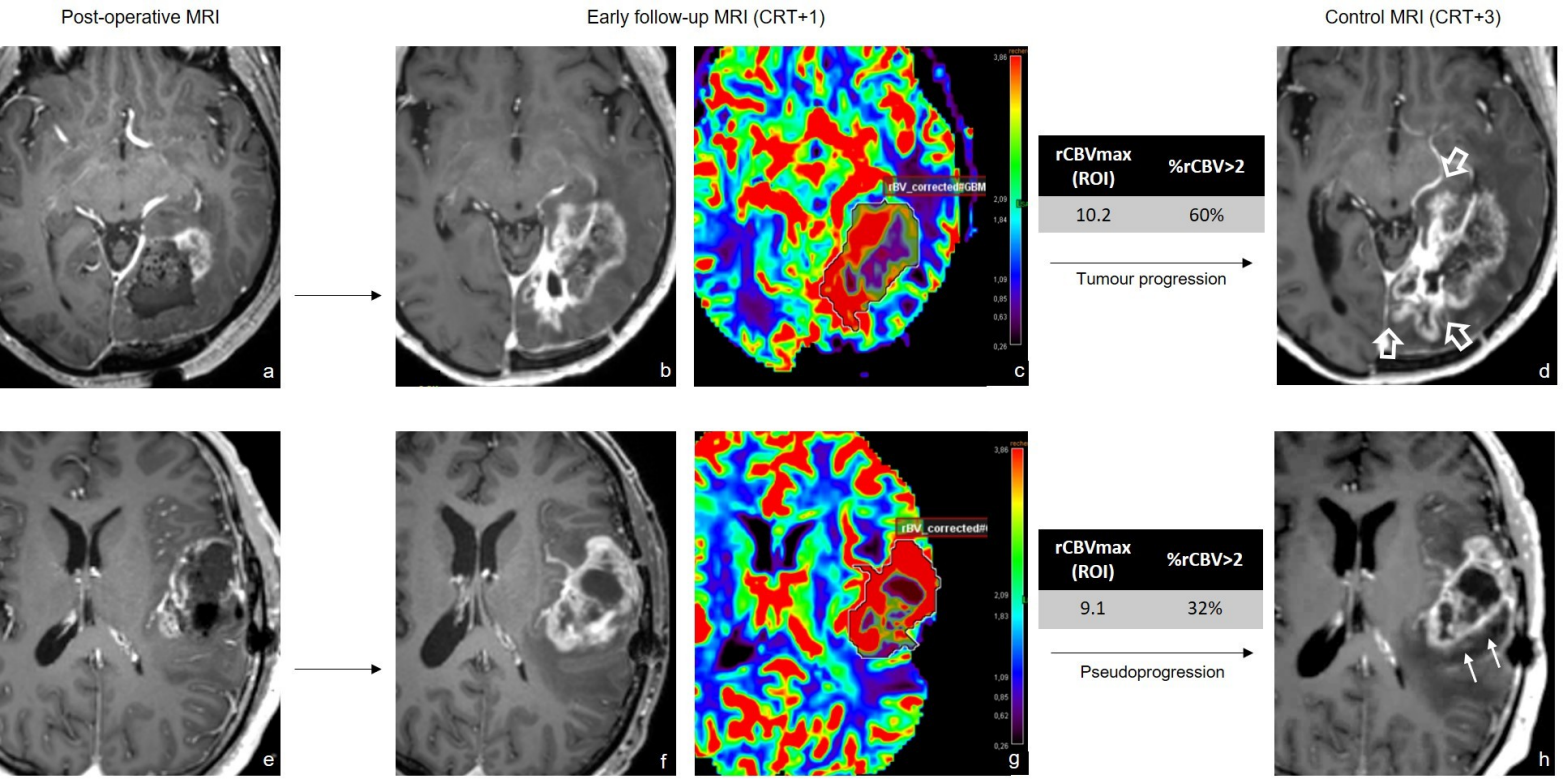
Longitudinal follow-up of two different glioblastomas with similar high rCBVmax (ROI) and different %rCBV>2. Axial contrast-enhanced T1-weighted images (CE-T1WI) of post-operative MRI (a and e), showing progressive lesions on CE-T1WI of CRT+1 MRI (b and f) with their corrected rCBV maps (c and g) and subsequent evolution on CRT+3 MRI (d and h). Top line (a-d) shows tumour progression (arrows) at CRT+3, calculated rCBVmax was 10.1 and %rCBV>2 was 60%. Bottom line (d-f) demonstrates regression of the lesion (thin arrows) indicative of pseudoprogression, despite high rCBVmax (9.1), %rCBV>2 was 32% and could have predicted pseudoprogression.

The ROC curve comparison showed a tendency for better diagnostic accuracy for %rCBV>2 (AUC = 0.909) compared to rCBVmax calculated with the hot-spot (AUC = 0.753; p = 0.11) or the volume method (AUC = 0.675; p = 0.06). When %rCBV>2 was under 41.5%, pseudoprogression could be diagnosed with 100% sensitivity and 71% specificity (Fig 4D). Fig 5 illustrates our main finding and shows two patients with progressive enhancing lesions on the RT+1 MRI, displaying similar rCBVmax but different %rCBV>2. Interestingly, the patient who displayed a high %rCBV>2 = 60% had a progressive tumour, while the other patient who displayed a low %rCBV>2 = 32% developed pseudoprogression.

The inter-observer agreement for quantitative measurement of %rCBV>2 was excellent (ICC = 0.88 [0.75–0.95]) as for pseudoprogression diagnosis with the 41.5% threshold (kappa = 0.75; [0.51–1]).

### Exclusion of the central necrotic component of the volume analysis of perfusion on the early follow-up MRI

**Fig 6:**
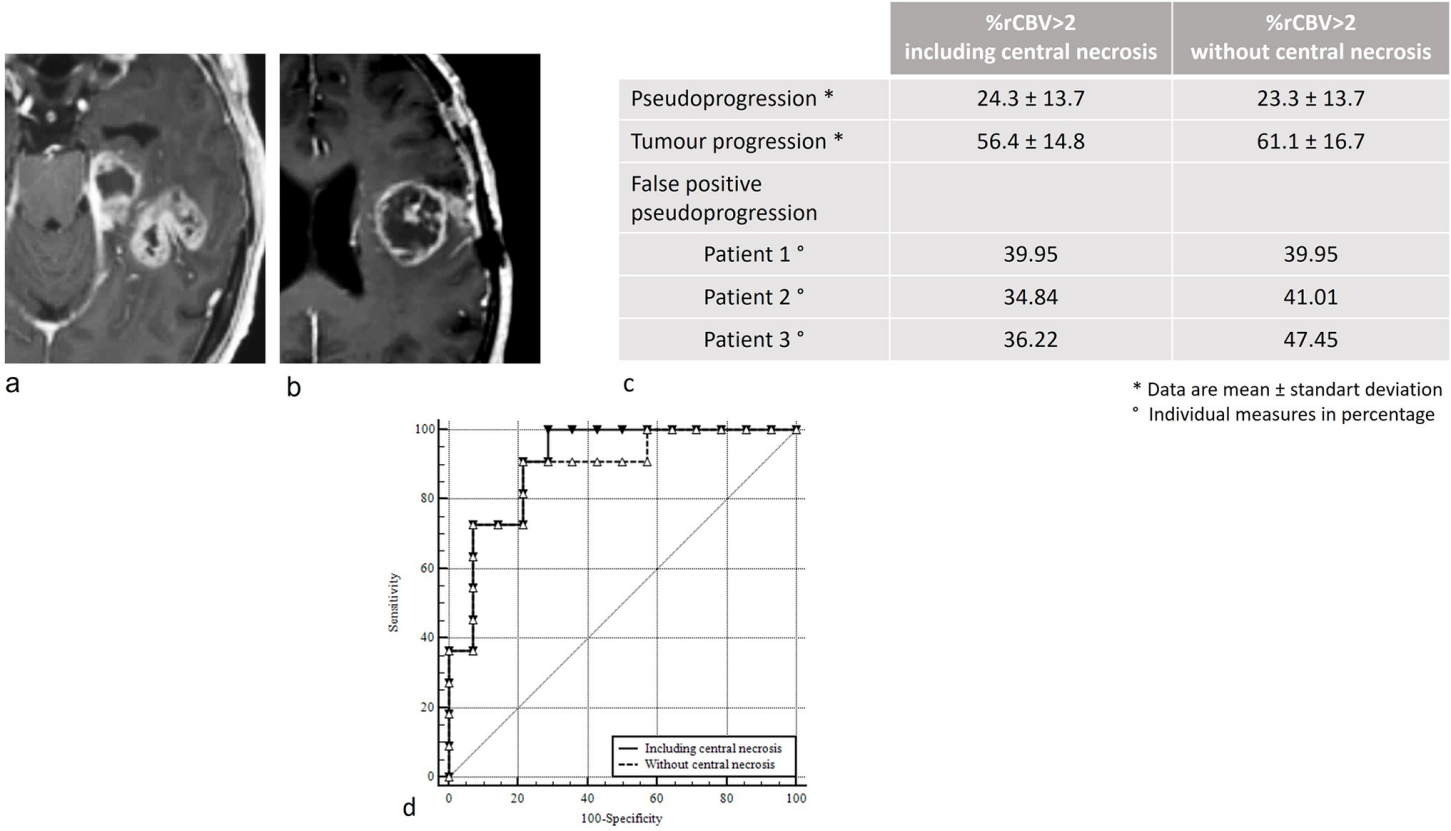
Pseudoprogression diagnosis accuracy of %rCBV>2 after exclusion of the central necrosis. Axial contrast-enhanced T1-weighted images (CE-T1WI) of early follow-up MRI of two morphologically different false positive (FP) pseudoprogression (PsP): (a) partially necrotic and (b) mostly necrotic. Table (c) presents %rCBV>2 mean measurements of %rCBV>2 in the PsP and tumour progression groups and individual measures for FP PsP patients, before and after exclusion of the central necrotic component from perfusion analysis and ROC curves comparison (d) for the diagnosis of PsP shows no statistical difference.

Including the central necrotic non-enhancing component of the lesions could have led to an underestimation of %rCBV>2 and a misdiagnosis of pseudoprogression, especially in case of highly necrotic lesions. On CRT+1 MRIs, we secondly segmented those necrotic parts and excluded them from the volume analysis. Pseudoprogression mean %rCBV>2 was 23.3 ± 13.7 and tumour progression mean %rCBV>2 was 61.1 ± 16.7. The new AUC for %rCBV>2 was 0.883 [0.692 to 0.976] (p = 0.507), showing no significant advantage in excluding necrosis from the analysis. Two of the 3 patients with tumour progression who were misdiagnosed as having pseudoprogression still displayed a %rCBV>2 under the 41.5% threshold (Fig 6).

## Discussion

From the volume analysis of DSC-PWI, we developed a parameter that assessed the fraction of hypervascularized tissue of an entire contrast-enhanced cerebral tumour: %rCBV>2. This fractional parameter helped to differentiate subsequent pseudoprogression from true progression of treated glioblastomas, even on the initial MRI (58% vs. 71%; p = 0.03) and with a higher confidence level on the early follow-up MRI performed 1 month after CRT (22% vs. 52%; p <0.001). On the early follow-up MRI, when a lesion had a %rCBV>2 under 41.5%, pseudoprogression could then be diagnosed with 100% sensitivity and 71% specificity.

Evaluation of the fraction of an entire contrast-enhanced lesion displaying a rCBV greater than 2, allowed us to assess the proportion of hypervascularization within this lesion. We assumed that this parameter would provide a better indication of the hypervascularization of heterogeneous tumours such as glioblastomas than measurements of a few selected points using the conventional hot-spot method. On the initial MRI, the conventional parameter rCBVmax was no different for glioblastomas that would subsequently develop pseudoprogression or tumour progression after CRT. However, the fractional parameter, %rCBV>2, was lower in the pseudoprogression group, potentially indicating a difference in the intrinsic neoangiogenesis and aggressiveness of the naive tumour. %rCBV>2, unlike rCBVmax, may be able to depict tumour sub-groups in an apparently homogeneous cohort. Those sub-groups could be linked to the genetic status of tumours, such as the MGMT status or other mutations, associated with a better therapeutic response and prognosis [21, 22]. In line with this, positive MGMT status was more frequent in our pseudoprogression group without reaching statistical significance.

As with previous studies, on early follow-up MRI (CRT+1), calculation of rCBVmax with the hot-spot method enabled us to differentiate between pseudoprogression and tumour progression groups [21, 23]. However, the high standard deviations of rCBVmax reflect the difficulty in clinical practice to confidently diagnose pseudoprogression and highlight the need for a more reliable method. In the pseudoprogression group, rCBVmax calculated with the volume method was substantially higher than with the hot-spot method at 7.3 and 3.4, respectively (p = 0.01). Pseudoprogression is heterogeneous and may contain highly vascularized portions of remaining tumour [11, 12]. Unlike the hot-spot method, which only assesses of a few points within the lesion causing a sampling bias, the volume method allows the perfusion to be evaluated over the entire volume and could allow detection of areas of remaining tumour with high neoangiogenesis. The co-existence of neoangiogenesis and necrosis weakens the capacity of any sampling method such as the hot-spot method or even stereotactic biopsy to diagnose pseudoprogression [6]. Volume analysis of MRI-perfusion data may be key to providing a better indication of heterogeneity.

A similar parameter, the perfusion MRI-fractional tumour burden (pMRI-FTB), defined as the percentage of tumour voxels relative to total lesion mask voxels, has already been correlated with the overall survival of progressive glioblastomas and used to differentiate treatment effect from tumour recurrence [24, 25]. The former study was based exclusively on histopathological examination, made on subtotal resection (60%) or stereotactic biopsy (40%), possibly misrepresenting the actual lesion. Additionally, even on one given sample, neuropathological diagnosis for suspected recurrence of glioblastomas varies dramatically (Kappa score 0.228), raising concerns about the use of histopathology as a gold standard [26]. Our results, mostly based on clinical and radiological follow-up, reinforce the fact that evaluation of the hypervascularized fraction of a treated glioblastoma could help when progression is questioned.

Unlike for pMRI-FTB and some previous studies [17, 24], we included the central necrotic component in the perfusion analysis. In this work, we aimed to develop a volume analysis that could be used in routine clinical practice by a radiologist without the need to use an advanced software. Sadly, automatic segmentation tools are not always available in clinical practice and segmentation of the enhancing part of glioblastomas can be tricky and time-consuming as necrosis is often ill-defined [27]. Besides, MRI analysis of the necrotic component may help in better characterisation of lesions as diffusion restriction in the non-enhancing necrotic centre of treated glioblastomas has been associated with radiation-induced coagulative necrosis [12, 28]. In another study, Dijkstra *et al*. showed that freeform ROIs that encompassed the whole tumour had a good diagnostic accuracy in differentiating low grade from high grade tumour [29]. In accordance with these results, we suggest that exclusion of the necrotic centre could be skipped from routine post-processing without preventing the distinction between pseudoprogression and tumour progression.

This study has some limitations. First, the retrospective and limited number of patients, which is common in most studies on pseudoprogression. To ensure a homogenous cohort, we used restrictive inclusion and exclusion criteria. For example, all perfusion data used were acquired on 3T systems since rCBV can be over-evaluated at 3T in comparison to 1.5T [30]. However, our results are consistent with previous publications and will add to the existing literature on pseudoprogression. Second, acquisition of DSC-MRI was not optimal at the time of the study. Indeed, our protocols did not follow the current recommendations as we used post-processing leakage-correction without a gadolinium pre-bolus dose, 45 dynamic acquisition time points and suboptimal flip angles [31, 32]. This could have resulted in an underestimation of CBV values, which could explain why our parameter lacked specificity for the diagnosis of pseudoprogression. However, it reached a perfect sensitivity. Then, as manual segmentation performed by experts is still considered the gold standard, we chose to rely on the neuroradiologist assessment of the tumour volume [33]. Although some authors demonstrated better reproducibility with semi-automatic segmentation in the measurement of perfusion parameters of glioblastomas, segmentation software remain rather reserved for research activities [27]. However, this type of study, showing the interest of a volume approach to evaluate perfusion parameters of glioblastomas, could encourage manufacturers to offer effective segmentation software more easily. Finally, the inter-observer variability assessment of %rCBV>2 was exclusively conducted on early follow-up MRI (CRT+1) by one second observer. Inter-observer agreement was excellent (ICC = 0.88), which is better than the variability of rCBVmax previously reported ranging from 0.37 to 0.71 [13, 19]. Moreover, authors have shown that manual segmentation and rCBVmax reproducibility considerably worsens after treatment, presuming that our reproducibility may have been even better if calculated on both initial and post-treatment MRIs [33, 34]. A dedicated inter- and intra-observer variability study should confirm the reliability of this fractional parameter.

## Conclusion

The fraction of hypervascularization in glioblastomas assessed by our fractional parameter %rCBV>2 was lower in patients that will subsequently develop pseudoprogression compared to true progression, on both initial and early follow-up MRIs. This parameter seems to better represent the heterogeneity of these enhancing lesions and better identify pseudoprogression than rCBVmax, conventionally calculated with the hot-spot method. In our cohort pseudoprogression could have been diagnosed on early follow-up MRI with 100% sensitivity and 71% specificity when %rCBV>2 was under 41.5%.
